# The Incremental Prognostic Value of the Clinical Residual SYNTAX Score for Patients With Chronic Renal Insufficiency Undergoing Percutaneous Coronary Intervention

**DOI:** 10.3389/fcvm.2021.647720

**Published:** 2021-04-15

**Authors:** Liqiu Yan, Peiyao Li, Yabin Wang, Dong Han, Sulei Li, Min Jiang, Xufen Cao, Feng Cao

**Affiliations:** ^1^The Second Medical Center & National Clinical Research Center for Geriatric Diseases, Chinese PLA General Hospital, Beijing, China; ^2^Department of Cardiology, Cangzhou Central Hospital, Hebei Medical University, Cangzhou, China; ^3^Department of Computer Science, Tsinghua University, Beijing, China; ^4^Artificial Intelligence Lab, Global Health Drug Discovery Institute, Beijing, China

**Keywords:** residual SYNTAX score, clinical residual SYNTAX score, coronary artery disease, percutaneous coronary intervention, chronic renal insufficiency

## Abstract

**Background:** The residual SYNTAX score (RSS) is considered a powerful prognostic indicator for determining a reasonable revascularization strategy in patients undergoing percutaneous coronary intervention (PCI), but the absence of clinical parameters is one of the limitations of RSS, especially in the chronic renal insufficiency (CRI) comorbidity setting. The present work aimed to investigate the incremental prognostic value of clinical residual SYNTAX score (CRSS) compared with RSS in CRI cases after PCI.

**Methods:** Totally 2,468 consecutive CRI cases who underwent PCI from January 2014 to September 2017 were included in this retrospective analysis. CRSS was obtained by multiplying RSS by the modified ACEF score. Individuals with CRSS >0 were considered to have incomplete revascularization and stratified by CRSS tertiles, the remaining cases constituted the complete revascularization (CR) group. The outcomes between these groups were compared.

**Results:** At a median follow-up of 3 years, compared with CR group, individuals with CRSS >12 showed elevated rates of all clinical outcomes, and those with CRSS ≤ 12 showed similar all-cause and cardiac mortality rates. In multivariable analysis, CRSS was a powerful independent predictive factor of all clinical outcomes. The net reclassification improvement levels of CRSS over RSS for all-cause and cardiac mortality rates were 10.3% (*p* = 0.007) and 16.4% (*p* < 0.001), respectively. Compared with RSS, CRSS markedly ameliorated all-cause and cardiac mortality risk stratification.

**Conclusions:** Compared with RSS, CRSS has incremental predictability for long-term all-cause and cardiac mortality in CRI cases following PCI.

## Introduction

The residual SYNTAX score (RSS) is considered an independent predictive factor of adverse outcomes among individuals undergoing percutaneous coronary intervention (PCI), and might aid in assessing a rational degree of revascularization (1–7). Our recent study demonstrated that RSS has stronger predictability than baseline SYNTAX score (SS) for unplanned revascularization (UR) and stroke, as well as major adverse cardiovascular and cerebrovascular events (MACCEs) in chronic renal insufficiency (CRI) cases undergoing PCI at the 3-year follow-up (8). However, the absence of clinical parameters in RSS represents one of the limitations in its ability to precisely risk stratify individuals with complex coronary artery disease (CAD). Previous studies have shown the prognostic value of clinical residual SYNTAX score (CRSS), which is derived as a product of RSS and modified ACEF (ACEF_CRCL_) score (3, 9). However, no previous studies have assessed the predictive potential of CRSS in CRI cases. CRI is a traditional risk factor for cardiovascular morbidity and mortality. It is associated with poor procedural success rate, increased complications and worse clinical outcomes, such as restenosis and stent thrombosis, after PCI (10, 11). Therefore, the goal of the current work was to determine the prognostic ability of CRSS comparatively to RSS in CRI cases.

## Methods

### Study Population

**Figure 1:**
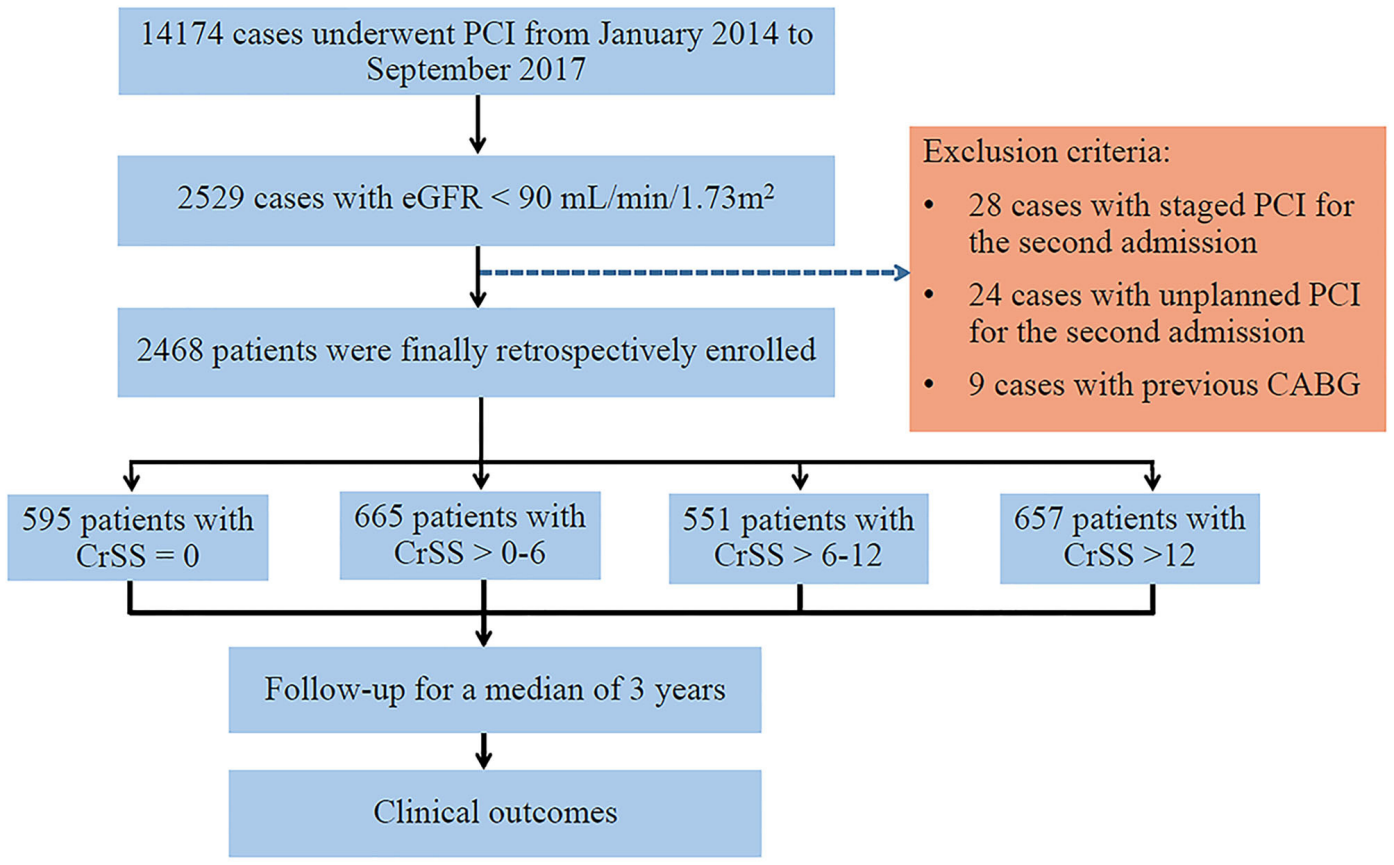
Study Flow Chart. CABG, coronary artery bypass graft; CrSS, clinical residual SYNTAX score; eGFR, estimated glomerular filtration rate; PCI, percutaneous coronary intervention.

A total of 14,174 consecutive patients were administered PCI between January 2014 and September 2017 in Cangzhou Central Hospital, Hebei Medical University. Among them, 2529 cases with an estimated glomerular filtration rate (EGFR) <90 mL/min per 1.73 m^2^ were consecutively enrolled. The simplified Modification of Diet in Renal Disease (MDRD) formula was used for EGFR assessment (12). Considering the SS can be used only in individuals with native CAD, nine patients with prior coronary artery bypass grafting (CABG), 28 with staged PCI and 24 with unplanned PCI for the second hospitalization were excluded. Finally, 2,468 individuals were assessed in this retrospective study (Figure 1). Ethical approval was obtained from the ethics committee of Cangzhou Central Hospital, Hebei Medical University. Each patient was asked to provide signed informed consent.

### Clinical Residual SYNTAX Score

CRSS was derived as follows: CRSS = (RSS) × (ACEF_CRCL_ score) (13). Baseline SS was calculated by visualization by two of three experienced interventionists who were well-trained in SS evaluation and blinded to patient grouping and outcomes. In case of disagreement, the third cardiologist provided an opinion, and a consensual decision was made after discussion (14). Subsequently, RSS was determined according to the remaining obstructive coronary disease treated by PCI (1). The ACEF_CRCL_ score was obtained as age/ejection fraction +1 point for every 10 mL/min decrease in CRCL below 60 mL/min per 1.73 m^2^ (maximum of six points).

### Study Endpoints

Complete revascularization (CR) was reflected by a post-PCI CRSS = 0. Individuals with incomplete revascularization (IR) were stratified according to CRSS tertiles. The primary endpoints included cardiac death (CD) and all-cause death (ACD). The secondary endpoints were myocardial infarction (MI), UR, stroke, and MACCEs (combining ACD, MI, stroke and UR). Revascularization was defined as UR for ischemic symptoms and events driven by CABG or PCI. The above endpoints were examined separately by 2 cardiologists, and any disagreement was solved by consensual discussion.

### Statistical Analysis

Continuous data were presented as mean ± SD, and compared by one-way ANOVA. Categorical data were expressed as *n* (%), and compared by the chi-square or Fisher's exact test. The Kaplan-Meier method was utilized for assessing clinical outcomes, with the log-rank test for comparison. The patients were considered to be at risk up to the last follow-up. Multivariable analysis was conducted by a Cox regression model utilizing the enter method. In addition to CRSS, variables related to adverse events were incorporated in the model for analysis. The Supremum test was employed to verify the proportional hazards assumption for various endpoints. By combining the ACEF_CRCL_ score with RSS, improvement in model performance, discrimination and risk classification was examined, by (a) comparatively evaluating the areas under the curves (AUCs) for the two nested models using the nonparametric method and (b) calculating net reclassification improvement (NRI) and integrated discrimination improvement (IDI) indices (15). Two-sided *p* < 0.05 was regarded as statistically significant. SPSS 24.0 (IBM Corp., Armonk, NY, USA) and R 3.6.0 were utilized for data analysis.

## Results

### Baseline Characteristics and Procedural Results

The values of RSS ranged from 0 to 44.5 (6.4 ± 6.6), and CRSS ranged from 0 to 130.2 (9.4 ± 12.6). CR was achieved in 595 patients (24.1%). The clinical and angiographic features of all cases after stratification by CRSS are summarized in Tables 1, 2. In comparison with individuals with CR, those with IR had increased rates of clinical comorbidities, including older age, diabetes, clinical presentation of MI, old MI, prior stroke, reduced EGFR, lower left ventricular ejection fraction, lower hemoglobin and higher fasting glucose (*p* < 0.05 or *p* < 0.001). Patients with IR had anatomically complex coronary disease, including higher baseline SS, three-vessel and left main disease, long lesions, bifurcations or trifurcations, aorto-ostial lesions, severe calcification and tortuosity, thrombus and chronic total occlusion lesions (all *p* < 0.001). Patients with IR had longer total length of implanted stents (*p* = 0.027) and increased target vessel number (*p* < 0.001).

**Table 1:** Baseline clinical characteristics.

	**CRSS = 0 (*n* = 595)**	**CRSS >0–6 (*n* = 665)**	**CRSS >6–12 (*n* = 551)**	**CRSS >12 (*n* = 657)**	***P*-Value**
Age, years	63.7 ± 9.3	63.0 ± 9.0	65.0 ± 8.2	68.2 ± 7.6	<0.001
Male	345 (58.0)	404 (60.8)	322 (58.4)	381 (58.0)	0.702
BMI, kg/m^2^	25.9 ± 3.4	25.9 ± 3.3	26.2 ± 3.3	26.0 ± 3.4	0.380
Diabetes	116 (19.5)	141 (21.2)	116 (21.2)	193 (29.4)	<0.001
Hypertension	376 (63.2)	443 (66.6)	387 (70.2)	452 (68.8)	0.058
Dyslipidemia	222 (37.3)	268 (40.3)	222 (40.3)	261 (39.7)	0.677
Current smoker	55 (9.2)	92 (13.8)	59 (10.7)	69 (10.5)	0.060
Prior MI	36 (6.1)	57 (8.6)	42 (7.6)	73 (11.1)	0.012
Previous PCI	77 (12.9)	96 (14.4)	71 (12.9)	78 (11.9)	0.583
Previous stroke	49 (8.2)	64 (9.6)	67 (12.2)	89 (13.5)	0.011
COPD	11 (1.8)	8 (1.2)	9 (1.6)	12 (1.8)	0.778
Clinical presentation					0.001
Stable angina	261 (43.9)	300 (45.1)	235 (42.6)	214 (32.6)	
Unstable angina	66 (11.1)	97 (14.6)	62 (11.3)	90 (13.7)	
NSTEMI	80 (13.4)	99 (14.9)	100 (18.1)	141 (21.5)	
STEMI	188 (31.6)	169 (25.4)	154 (27.9)	212 (32.3)	
eGFR, ml/min	76.3 ± 11.8	78.5 ± 9.1	76.4 ± 11.4	70.1 ± 16.5	<0.001
Renal dysfunction					
60 ≤ EGFR <90	538 (90.4)	644 (96.8)	507 (92.0)	484 (73.7)	<0.001
30 ≤ EGFR <60	54 (9.1)	20 (3.0)	42 (7.6)	155 (23.6)	
EGFR <30	3 (0.5)	1 (0.2)	2 (0.4)	18 (2.7)	
LVEF, %	60.5 ± 9.6	61.1 ± 8.1	61.3 ± 9.2	56.5 ± 10.5	<0.001
LVEDD (mm)	47.8 ± 6.7	47.7 ± 5.6	47.9 ± 6.0	49.0 ± 6.5	0.025
Baseline laboratory					
Hemoglobin (mg/dL)	13.3 ± 1.7	13.4 ± 1.6	13.1 ± 1.6	12.9 ± 1.8	<0.001
Creatinine (mg/dL)	0.97 ± 0.23	0.94 ± 0.17	0.97 ± 0.26	1.08 ± 0.49	<0.001
Fasting glucose (mg/dL)	136.1 ± 66.7	133.7 ± 59.6	134.7 ± 59.3	150.7 ± 77.5	<0.001
Total cholesterol (mg/dL)	170.9 ± 39.3	174.3 ± 43.3	172.8 ± 40.8	175.7 ± 42.5	0.295
TG (mg/dL)	154.6 ± 106.5	164.7 ± 124.0	162.6 ± 110.5	160.8 ± 100.4	0.419
HDL (mg/dL)	37.3 ± 9.3	37.0 ± 8.6	36.3 ± 8.2	36.3 ± 8.9	0.110
LDL (mg/dL)	99.3 ± 33.0	101.5 ± 32.3	100.5 ± 30.9	102.6 ± 30.8	0.300

*Data are the mean ± SD or n (%). CRSS, clinical residual SYNTAX score; BMI, body mass index; MI, myocardial infarction; PCI, percutaneous coronary intervention; COPD, chronic obstructive pulmonary disease; NSTEMI, non ST-segment elevation myocardial infarction; STEMI, ST-segment elevation myocardial infarction; EGFR, estimated glomerular filtration rate; LVEF, left ventricular ejection fraction; LVEDD, left ventricular end diastolic diameter; TG, triglyceride; HDL, high density lipoprotein; LDL, low density lipoprotein*.

**Table 2:** Angiographic and procedural characteristics.

	**CRSS = 0 (*n* = 595)**	**CRSS >0–6 (*n* = 665)**	**CRSS >6–12 (*n* = 551)**	**CRSS >12 (*n* = 657)**	***P*-Value**
**CAD extension**					<0.001
1-vessel disease	428 (71.9)	74 (11.1)	17 (3.1)	9 (1.4)	
2-vessel disease	142 (23.9)	360 (54.1)	216 (39.2)	125 (19.0)	
3-vessel disease	25 (4.2)	231 (34.7)	318 (57.7)	523 (79.6)	
Left main disease	17 (2.9)	28 (4.2)	21 (3.8)	93 (14.2)	<0.001
**Lesion anatomical characteristics**					
Lesion length > 20 mm	219 (36.8)	356 (53.5)	309 (56.1)	440 (67.0)	<0.001
Bifurcation or trifurcation	72 (12.1)	182 (27.4)	161 (29.2)	216 (32.9)	<0.001
Aorto-ostial lesion	5 (0.8)	8 (1.2)	13 (2.4)	30 (4.6)	<0.001
Heavy calcification	17 (2.9)	27 (4.1)	46 (8.3)	132 (20.1)	<0.001
Severe tortuosity	15 (2.5)	37 (5.6)	32 (5.8)	67 (10.2)	<0.001
Thrombus	117 (19.7)	74 (11.1)	67 (12.2)	73 (11.1)	<0.001
Chronic total occlusions	55 (9.2)	65 (9.8)	51 (9.3)	186 (28.3)	<0.001
Target vessel number	1.29 ± 0.55	1.34 ± 0.58	1.29 ± 0.51	1.19 ± 0.45	<0.001
**Target lesion location**					
LM	14 (2.4)	29 (4.4)	19 (3.4)	9 (3.4)	0.008
LAD	377 (63.4)	449 (67.5)	273 (49.5)	231 (35.2)	<0.001
LCX	156 (26.2)	163 (24.5)	177 (32.1)	202 (30.7)	0.008
RCA	220 (37.0)	250 (37.6)	240 (43.6)	337 (51.3)	<0.001
**Procedural characteristics**					
Stent per patient	1.70 ± 1.05	1.81 ± 0.99	1.75 ± 0.88	1.69 ± 0.86	0.072
Total length of stent, mm	45.0 ± 32.2	50.0 ± 30.7	48.1 ± 28.2	47.0 ± 27.9	0.027
Stent length >100 mm	40 (6.7)	54 (8.1)	34 (6.2)	39 (5.9)	0.398
Mean stent diameter, mm	3.03 ± 0.45	2.98 ± 0.46	2.96 ± 0.45	2.90 ± 0.46	<0.001
Minimum stent diameter, mm	2.95 ± 0.45	2.88 ± 0.45	2.84 ± 0.43	2.81 ± 0.45	<0.001
Maximum stent diameter, mm	3.18 ± 0.45	3.14 ± 0.47	3.08 ± 0.46	3.03 ± 0.49	<0.001
Primary PCI	84 (14.1)	47 (7.1)	65 (11.8)	62 (9.4)	<0.001
Baseline SYNTAX score	8.88 ± 5.90	11.98 ± 5.84	15.15 ± 5.66	21.18 ± 7.16	<0.001
Baseline SYNTAX score					<0.001
Low (<22)	576 (96.8)	614 (92.3)	480 (87.1)	388 (59.1)	
Median (22–32)	19 (3.2)	48 (7.2)	65 (11.8)	218 (33.2)	
High (>32)	0 (0.0)	3 (0.5)	6 (1.1)	51 (7.8)	
Residual SYNTAX score	0	3.33 ± 1.41	8.85 ± 1.64	24.59 ± 15.61	<0.001
Delta SYNTAX score	8.88 ± 5.90	9.04 ± 5.78	7.66 ± 5.70	6.58 ± 5.01	<0.001

*Data are the mean ± SD or n (%). CRSS, clinical residual SYNTAX score; CAD, coronary artery disease; LM, left main; LAD, left anterior descending artery; LCX, left circumflex; RCA, right coronary artery; PCI, percutaneous coronary intervention*.

### Clinical Outcomes

**Figure 2:**
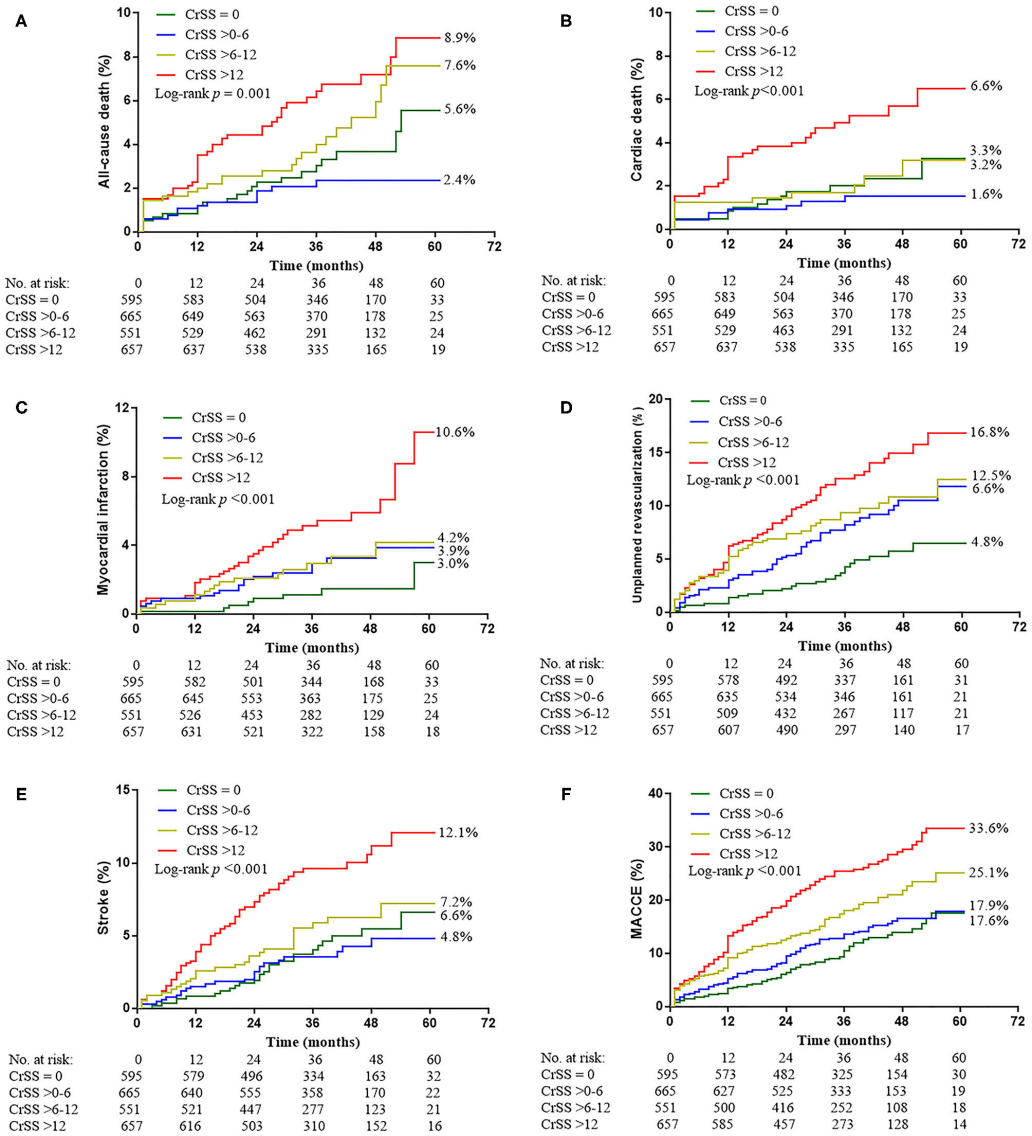
Kaplan-Meier Curves Showing Event Rates Stratified by the CRSS Through 5 Years. **(A)** All-cause death. **(B)** Cardiac death. **(C)** Myocardial infarction. **(D)** Unplanned revascularization. **(E)** Stroke. **(F)** Major adverse cardiovascular and cerebrovascular events (MACCE).

**Figure 3:**
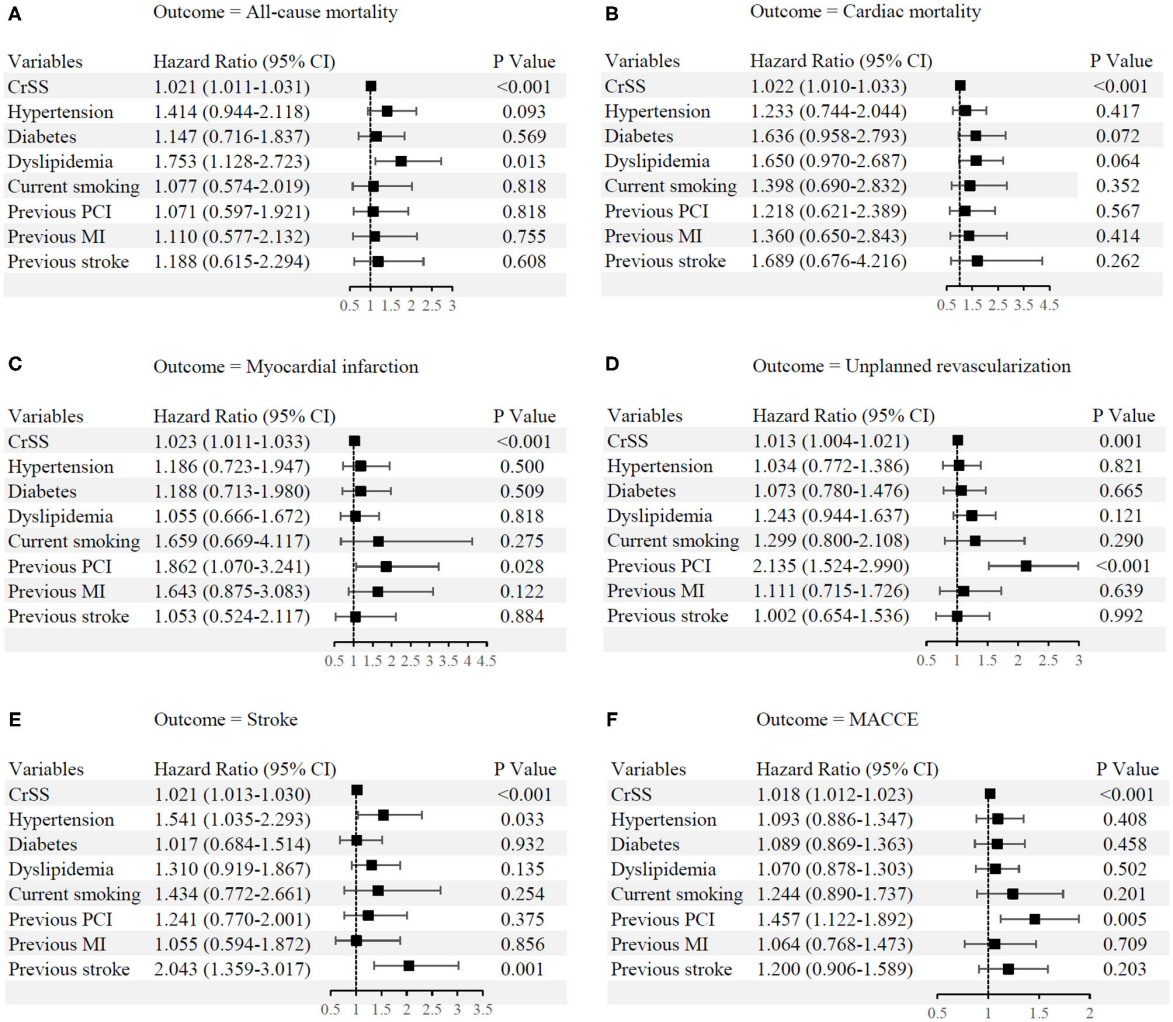
Independent predictors of long-term clinical outcomes. **(A)** All-cause mortality. **(B)** Cardiac mortality. **(C)** Myocardial infarction. **(D)** Unplanned revascularization. **(E)** Stroke. **(F)** Major adverse cardiovascular and cerebrovascular events. CI, confidence interval; CRSS, clinical residual SYNTAX score; MACCE, major adverse cardiovascular and cerebrovascular events; MI, myocardial infarction; PCI, percutaneous coronary intervention.

There were 2,425 patients (98.3%) who completed the median 3-year (1.5~5 years) follow-up. Clinical outcomes stratified based on CRSS are depicted in Table 3 and Figure 2. Patients with CRSS >12 had increased 5-year cumulative incidence rates of adverse events, including ACD (8.9%), CD (6.5%), MI (10.6%), UR (16.8%), stroke (12.1%) and MACCEs (33.6%) (all *p* ≤ 0.001). Subjects with CRSS ≤ 12 had ACD, CD, MI, and stroke rates comparable to those with CR. Multivariable Cox regression analysis demonstrated that CRSS independently predicted all clinical outcomes at a follow-up of 3 years (Figure 3).

**Table 3:** Five-Year cumulative incidence of adverse events according to CRSS.

	**CRSS, 0 (a)**	**CRSS > 0–6 (b)**	**CRSS > 6–12 (c)**	**CRSS > 12 (d)**	***P*****-Value**						
					**Trend**	a vs. b^*^	a vs. c^*^	a vs. d^*^	b vs. c^*^	b vs. d^*^	c vs. d^*^
All-cause death	5.6% (33)	2.4% (16)	7.6% (42)	8.9% (58)	0.001	0.195	0.235	0.01	0.015	<0.001	0.168
Cardiac death	3.3% (20)	1.6% (10)	3.2% (18)	6.5% (43)	<0.001	0.282	0.929	0.006	0.259	<0.001	0.01
Myocardial infarction	3.0% (18)	3.9% (26)	4.2% (23)	10.6% (70)	<0.001	0.058	0.049	<0.001	0.899	0.017	0.036
Unplanned revascularization	6.5% (39)	11.9% (79)	12.5% (69)	16.8 (110)	<0.001	0.005	0.001	<0.001	0.448	0.007	0.081
Stroke	6.6% (39)	4.8% (32)	7.2% (40)	12.1% (80)	<0.001	0.647	0.301	<0.001	0.139	<0.001	0.01
MACCE	17.6% (105)	17.9% (119)	25.1% (138)	33.6% (220)	<0.001	0.216	<0.001	<0.001	0.019	<0.001	0.002

*Event rates are Kaplan-Meier estimates, % (n)*.

* *Adjusted significance level is 0.008. CRSS, clinical residual SYNTAX score, MACCE, major adverse cardiovascular and cerebrovascular events*.

### Discriminative and Predictive Capability of CRSS and RSS

**Figure 4:**
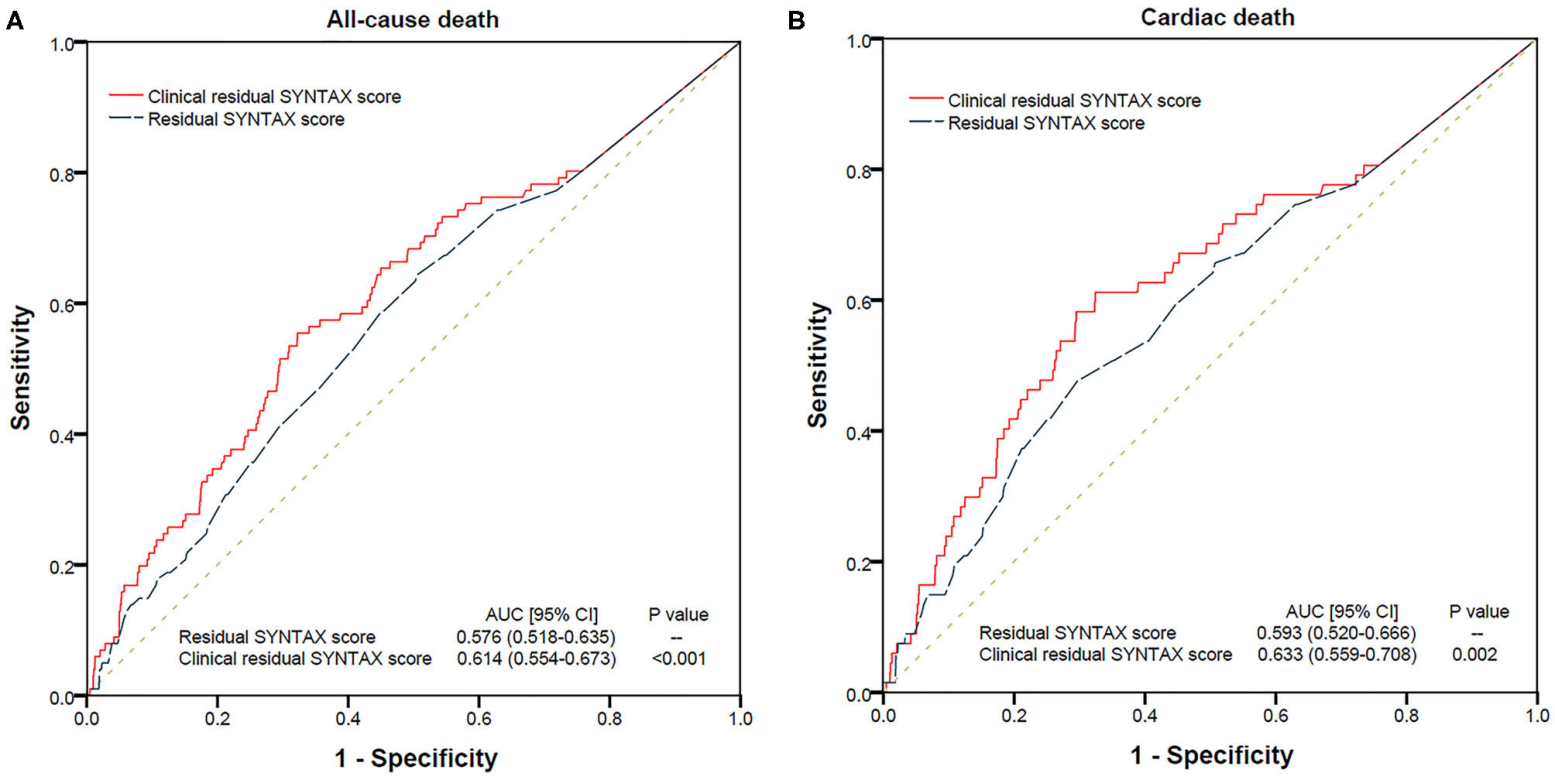
Comparison of ROC Curves between RSS and CRSS for Predicting Long-term All-cause **(A)** and Cardiac **(B)** mortality. AUC, area under the curve; CRSS, clinical residual SYNTAX score; ROC, receiver operating characteristic; RSS, residual SYNTAX score.

Receiver operating characteristic (ROC) curve analysis revealed CRSS was significantly associated with 3-year all-cause and cardiac mortality rates, MI, UR, stroke and MACCEs (all *p* < 0.001). A CRSS cutoff of 9.8 had a predictive value for all-cause and cardiac mortality (Table 4). CRSS demonstrated greater performance for all-cause and cardiac mortality as well as MACCEs in comparison with RSS (Table 5, Figure 4).

**Table 4:** ROC curve analysis of CRSS regarding long-term clinical outcomes.

	**AUC**	***P*-Value**	**Optimal Cutoff**	**Sensitivity (%)**	**Specificity (%)**
All-cause death	0.614	<0.001	9.8	55.5	67.7
Cardiac death	0.633	<0.001	9.8	61.2	67.6
Myocardial infarction	0.620	<0.001	11.2	48.7	71.8
Unplanned revascularization	0.588	<0.001	5.6	64.8	50.6
Stroke	0.609	<0.001	12.4	43.5	75.5
MACCE	0.609	<0.001	9.7	49.9	69.0

*ROC, receiver operating characteristic; CRSS, clinical residual SYNTAX score; AUC, area under the curve; and MACCE, major adverse cardiovascular and cerebrovascular events*.

**Table 5:** Comparison of ROC curves between RSS and CRSS.

		**AUC**	**95% CI**	***P*-Value**
All-cause death	RSS	0.576	0.518–0.635	–
	CRSS	0.614	0.554–0.673	<0.001
Cardiac death	RSS	0.593	0.520–0.666	–
	CRSS	0.633	0.559–0.708	0.002
Myocardial infarction	RSS	0.610	0.548–0.672	–
	CRSS	0.620	0.557–0.682	0.402
Unplanned revascularization	RSS	0.589	0.550–0.628	–
	CRSS	0.588	0.550–0.627	0.867
Stroke	RSS	0.598	0.548–0.649	–
	CRSS	0.609	0.557–0.660	0.178
MACCE	RSS	0.596	0.567–0.626	–
	CRSS	0.609	0.579–0.639	0.006

*ROC, receiver operating characteristic; RSS, residual SYNTAX score; CRSS, clinical residual SYNTAX score; AUC, area under the curve; CI, confidence interval; MACCE, major adverse cardiovascular and cerebrovascular events*.

Furthermore, CRSS markedly ameliorated risk stratification for all-cause and cardiac mortality. The NRI of CRSS vs. RSS for all-cause and cardiac mortality were 10.3% (*p* = 0.007) and 16.4% (*p* < 0.001), respectively. However, the IDI indices of CRSS vs. RSS for all-cause (*p* = 0.840) and cardiac mortality (*p* = 0.516) were not statistically significant (Table 6).

**Table 6:** Statistics for model improvement of CRSS over RSS for All-cause and cardiac mortality.

	**Model performance**		**Risk reclassification**		**Discrimination**	
	**Δ AUC**	***P*-Value**	**NRI (95% CI)**	***P*-Value**	**IDI (95% CI)**	***P*-Value**
All-cause death	0.038	<0.001	0.103 (0.029–0.181)	0.007	0.002 (−0.018–0.022)	0.840
Cardiac death	0.040	0.002	0.164 (0.081–0.248)	<0.001	0.009 (−0.036–0.018)	0.516

*CRSS, clinical residual SYNTAX score; RSS, residual SYNTAX score; AUC, area under the curve; CI, confidence interval; NRI, net reclassification improvement; IDI, integrated discrimination improvement*.

## Discussion

We comprehensively investigated the predictive value of CRSS for long-term patient outcomes in CRI cases after PCI. Our findings indicate that elevated CRSS is a representative marker of increased cardiac-kidney comorbidities and more anatomically complex disease. CRSS is a strong independent predictor of long-term adverse events such as all-cause and cardiac mortality. Furthermore, CRSS is superior to RSS in predicting long-term all-cause and cardiac mortality in CRI cases following PCI.

In this study, CR was achieved in only 24.1% of patients, which was obviously lower than previously reports (1–4, 16) but corroborating a recent study in which CR was achieved in 20.8% and 28.2% of CKD and non-CKD patients, respectively (17). CRI cases show higher rates of cardiovascular risk factors and comorbidities, anatomically complex disease, and complications associated with invasive procedures, as well as lower procedural success rates, and worse outcomes (10, 11). CRI cases have elevated risk of developing contrast-related acute kidney injury following PCI (18). Therefore, it is difficult for cardiologists to treat individuals with highly complex CAD and CRI. Furthermore, the current study demonstrated that patients with older age, increased clinical cardiac-kidney comorbidities and more anatomically complex disease, as quantified by CRSS, tended to have IR, corroborating previous reports (3, 9).

The RSS, which was first developed by Stone in a large cohort of acute coronary syndrome (ACS) cases, is considered an important tool for quantifying and risk-stratifying the degree and complexity of residual stenosis following PCI, with a strong independent predictive value for clinical outcomes (1). Its prognostic value has been fully validated in multivessel CAD cases (7, 16), individuals with unprotected left main disease (19), primary PCI cases (6, 20, 21), complex CAD after treatment with second-generation drug-eluting stents (4) and all-comers (3, 5). Our recent study also demonstrated that RSS can help assess reasonable levels of revascularization and ameliorate predictability accuracy in comparison with baseline SS for UR, stroke, and MACCEs for CRI cases after PCI (8). However, the absence of relevant clinical parameters is the main limitation of RSS. Previous studies have demonstrated the incremental prognostic value of scoring systems simultaneously including anatomic and clinical features in comparison with strictly anatomic SS (22). The clinical SS, which combines clinical variables (ACEF_CRCL_ score) with SS, showed superiority over SS or the ACEF_SCR_ score alone in predicting 5-year death and MACCEs in complex CAD after PCI (13).

Park and colleagues firstly described CRSS in patients included in a large, multicenter and all-comer PCI registry, and demonstrated that it has better predictive value for 1-year ACD and target lesion failure after PCI compared with RSS (3). Recently, Song and co-workers assessed the prognostic value of CRSS in a large-sample study examining real-world patients, and the results showed that CRSS has improved predictability of 2-year mortality than the anatomic RSS and SYNTAX revascularization index (9). However, few reports have investigated the predictive value of CRSS in CRI cases.

We first examined the predictive value of CRSS in CRI cases after PCI. In comparison with individuals with CR, those with CRSS >12 had progressively increasing adverse long-term outcomes such as all-cause and cardiac mortality; those with CRSS ≤ 12 had comparable long-term ACD, CD, MI and stroke rates. In multivariable analysis, CRSS was a strong independent predictive factor of all long-term outcomes. Furthermore, CRSS remarkably ameliorated all-cause and cardiac mortality risk stratification, suggesting that compared with RSS, CRSS has greater performance and risk classification ability for long-term all-cause and cardiac mortality in CRI cases. At the same time, this study showed RSS had better predictability accuracy for UR than CRSS, indicating the progression of residual coronary lesions is the main cause of UR. These findings suggest that clinicians should strengthen clinical management, follow-up and secondary prevention for high-risk patients with high CRSS to improve clinical outcomes.

## Limitations

Although this study was the first report to validate CRSS in CRI cases undergoing PCI, it had multiple limitations. First, participants were enrolled in a single center, which may lead to selection bias. Secondly, kidney function was examined by serum creatinine-derived EGFR rather than directly measuring kidney function, including iothalamate clearance. Furthermore, patients with CRI have a high risk of developing contrast-induced nephropathy (CIN), which is a strong predictor of poor clinical outcomes post-PCI. We did not evaluate the prognostic effect of CIN in this study. Finally, the functional assessment is paramount in guiding the clinical practice and improving clinical outcomes in patients undergoing PCI (23). However, we did not utilize fractional flow reserve to physiologically evaluate ischemia in vessels with residual disease because of the invasive nature and elevated cost of the procedure, and CR definition was only based on RSS. Further prospective multiple-center randomized studies are warranted for improved evaluation.

## Conclusions

In a large-size study including real-world CRI cases following PCI, CRSS independently predicted long-term all-cause and cardiac mortality, MI, UR, stroke and MACCEs, and had improved predictive value for long-term all-cause and cardiac mortality compared with RSS.

## Data Availability Statement

The original contributions presented in the study are included in the article/supplementary material, further inquiries can be directed to the corresponding author/s.

## Ethics Statement

The studies involving human participants were reviewed and approved by the ethics committee of Cangzhou Central Hospital, Hebei Medical University. The patients/participants provided their written informed consent to participate in this study. Written informed consent was obtained from the individuals for the publication of any potentially identifiable images or data included in this article.

## Author Contributions

LY, X-FC, and FC provided the conception of the idea for the study. LY, YW, DH, and FC contributed to the development of the methodology and wrote the manuscript. LY and PL analyzed the acquired data. SL and MJ were responsible for the interpretation of statistical results. FC revised the manuscript. All authors contributed to the article and approved the submitted version.

## Conflict of Interest

The authors declare that the research was conducted in the absence of any commercial or financial relationships that could be construed as a potential conflict of interest.
